# Environmental Risk Factors for Endometriosis: An Umbrella Review of a Meta-Analysis of 354 Observational Studies With Over 5 Million Populations

**DOI:** 10.3389/fmed.2021.680833

**Published:** 2021-10-25

**Authors:** Ye Zhang, Ning-Ye Ma

**Affiliations:** Department of Obstetrics and Gynecology, Shengjing Hospital of China Medical University, Shenyang, China

**Keywords:** endometriosis, meta-analysis, observational study, risk factor, umbrella review

## Abstract

**Background:** The association between a diverse array of environmental risk factors and the risk of endometriosis is contradictory.

**Objective:** To summarize the evidence of associations between environmental risk factors and the risk of endometriosis.

**Methods:** Databases such as PubMed, EMBASE, Web of Science, and ClinicalTrial.gov were systematically searched in June 2020. Meta-analyses of observational studies investigated any environmental exposure (non-genetic) and endometriosis risk. For each article, we estimated the summary effect size, 95% CIs, and the 95% prediction interval (PI). We also estimated the between-study heterogeneity expressed by *I*^2^, evidence for small-study effects, and evidence of excess significance bias.

**Results:** About 12 eligible articles (featuring 143,422 cases and 5,112,967 participants) yielded data on 40 unique environmental risk factors, including life styles (*n* = 16), reproductive factors (*n* = 3), early life factors (*n* = 4), and a range of other risk factors [e.g., phthalate metabolites, endocrine-disrupting chemicals, and body mass index (BMI)]. About 25 of these 40 associations (62.5%) were statistically significant (*p* < 0.05) under random-effects models. Evidence for an association was indicated for alcohol intake [relative risk (RR): 1.25; 95% CI: 1.11–1.41] and the exposure to endocrine disruptor chemicals (EDCs) (RR: 1.41; 95% CI: 1.23–1.60) while 15 associations presented only weak evidence.

**Conclusions:** Our analyses showed that alcohol intake and exposure to endocrine-disrupting chemicals may be potential risk factors for endometriosis and supported by suggestive epidemiological evidence. However, it was evident that there was substantial heterogeneity and/or bias between the different studies featured in various meta-analyses included in this review; therefore, the outcomes of our analysis should be interpreted cautiously.

## Introduction

Endometriosis is a chronic condition associated with pelvic pain, dyspareunia, and infertility and is thought to affect 6–10% of women of reproductive age (1). A recent report have demonstrated that all burden estimates of endometriosis have decreased on a global basis, however, the incidence, prevalence, and the number of years of life lived with disability, associated with this disease, exhibited an increasing trend in countries with a high sociodemographic index between 1990 and 2017 (2). These data indicate that countries with a high sociodemographic index should continue to focus on reducing the disease burden associated with endometriosis as a matter of priority.

Biologically, endometriosis is an estrogen-dependent, chronic, and inflammatory gynecological condition that is characterized by the proliferation of a functional endometrial tissue that develops outside the uterine cavity (3). In addition, it is suggested that the development of endometriosis is determined by the complex interplay and composite effects of both genetic and environmental risk factors. Families of genes associated with the immune system and inflammatory pathways, cell adhesion, and extracellular matrix remodeling have been reported to be differentially expressed when comparing between women with and without endometriosis (4, 5). As a common environmental risk factor, endocrine disruptor chemicals (EDCs) are widely present in the environment and food chains. EDCs could affect the dynamic balance of sex hormones and mediate the innate immune cell dysregulation, which may play an important role in the pathogenesis of endometriosis (6–8). In addition, dietary intake may also influence the development of endometriosis. Alcohol intake could increase the level of estrogen in blood circulation and induce a variety of cells to produce proinflammatory cytokines, which may be related to the pathogenesis of endometriosis (9). However, although previous epidemiological studies have suggested that several risk factors (e.g., diethylstilbestrol exposure, low birth weight, and early age at menarche) were associated with the risk of endometriosis (10), there is a clear lack of well-established and modifiable risk factors for this disease. Notably, there is still no conclusive evidence for these potential risk factors with respect to either the association itself or its direction. This is because several existing publications have yielded contradictory findings.

To the best of our knowledge, there has been no systematic effort to summarize and critically appraise this body of existing evidence. Therefore, we conducted the first umbrella review of the evidence arising from existing systematic reviews and meta-analyses of observational studies to provide an overview of the breadth, strength, and validity of the reported associations between a diverse array of risk factors and the risk of endometriosis. We summarize the risk factors that have been associated with endometriosis in previous meta-analyses, assess the quality of the methodology used, evaluate the evidence for diverse bias, and determine which of the associations are supported through convincing epidemiological evidence.

## Methods

We followed a standardized method and reported our findings in accordance with the recommendations put forward by the Preferred Reporting Items for Systematic Reviews and Meta-Analyses (Supplementary Table 1) and Meta-analyses of Observational Studies in Epidemiology recommendations (Supplementary Table 2) (11, 12). Our study protocol was registered with PROSPERO (No: CRD42020200094).

### Search Strategy

We performed an umbrella review (i.e., a systematic collection and assessment of multiple systematic reviews and meta-analyses published on a specific Research Topic) focused on the risk factors for endometriosis. We systematically searched the PubMed, EMBASE, Web of Science, and ClinicalTrial.gov databases from inception to June 30, 2020, to identify systematic reviews or meta-analyses of observational studies that were performed to examine the associations of environmental (non-genetic) factors and biomarkers with the risk of endometriosis with no restrictions. We used the following search strategy: (endometriosis) and (meta-analysis or systematic review) (as shown in Supplementary Table 3). In addition, we performed a manual search of the reference lists of all eligible retrieved publications. Two authors independently screened the titles, abstracts, and full-text articles for eligibility, and discrepancies were resolved through a consensus.

### Selection and Exclusion Criteria

Publications were initially screened on the basis of the title and by reading the abstract. The full texts of potentially eligible publications were then scrutinized by the two independent investigators (YZ and N-YM). Any disagreement was solved through a discussion. We considered the publications that were meta-analyses of epidemiological studies (case-control, cohort, cross-sectional, and ecological studies) that were conducted to investigate any environmental exposure (non-genetic) and the risk of endometriosis. If an article described a separate meta-analysis of more than one environmental risk factor, we included each of these factors separately. Furthermore, if there was more than one meta-analysis on the same association, we kept the one with the largest number of primary studies included. If meta-analyses on the same association included the same number of primary studies, we kept one with the largest amount of prospective data.

The publications that investigated pure genetic markers of endometriosis were excluded because they did not fall into the remit of this study. Trials were not available for our specific research question. We excluded the systematic reviews that did not feature quantitative analysis, meta-analyses based on individual data without a systematic review, or the articles that included animal trials or laboratory studies. We also excluded the systematic reviews or meta-analyses that lacked study-specific data (risk estimates, the number of cases and controls, or the number of the total study population).

### Data Extraction

For each eligible meta-analysis, we extracted the first author's name, journal name, publication year, the number of studies included, study population (general, mixed, or not report), environmental risk factors, type of effect metric in meta-analyses, and level of comparison. Also, we extracted information from each primary study used in meta-analyses, including the first author, publication year, study design (cross-sectional, case-control, or cohort), the number of cases and controls (for case-control studies), total participants or person-years (for cohort studies), risk estimates, and 95% CIs. We extracted the most fully adjusted risk estimates (odds ratio [OR], relative risk [RR], incident risk ratio [IRR], or standardized mean difference [SMD]) and 95% CIs. We also extracted information related to dose-response relationships from all meta-analyses. In practice, the measures of effect yield similar estimates because endometriosis is a rare occurrence. Two independent investigators (YZ and N-YM) extracted the data from eligible publications. In the case of discrepancies, the final decision was made through a discussion.

### Statistical Analysis

#### Estimation of Summary Effects and Heterogeneity

For each meta-analysis, we calculated the summary effect size, along with 95% CIs and the values of *p*, using both fixed- and random-effects models (13). Between-study heterogeneity was assessed with the *I*^2^ statistic (14). We also assessed the uncertainty surrounding heterogeneity estimates by calculating 95% CIs and the values of *p* (15). *I*^2^ values of 50% or more were considered to represent high levels of heterogeneity, whereas values exceeding 75% were considered to represent very high levels of heterogeneity.

#### Estimation of Prediction Intervals (PIs)

We calculated 95% PIs for the random-effects estimates to account for between-study heterogeneity and to represent the possible range in which the risk estimates of new studies might lie (16).

#### Assessment of Small-Study Effects

We calculated the SE of the effects associated with the largest data set (with the lowest SE) for each of the included meta-analyses. If the SE was <0.10, then the 95% CI would be <0.20 (which is less than the magnitude of small effect size). Egger's regression asymmetry test was also used to determine small-study effects. The value of *p* < 0.10 arising from Egger's test and a summary effect size larger than the effect size of the largest study were considered to represent evidence for small-study effects (17).

#### Evaluation of the Excess Significance

We calculated excess significance bias by investigating whether the observed (O) number of nominally significant findings was significantly different from the expected (E) number of statistically significant studies. To do this, we performed a chi-squared test to compare the difference between O and E (18). The effect size of the largest study in each meta-analysis was used to determine the power estimates for each component of a particular study using a non-central *t* distribution (19). Excess statistical significance for a single meta-analysis was determined with the value of *p* < 0.10 and if O > E (18). Statistical analyses were conducted using the Stata version 12.0 software (Stata Corp, College Station, TX, USA) and all values of *p* were two-tailed.

### Assessment of Methodological Quality

Two independent investigators (YZ and N-YM) assessed the methodological quality for each included systematic review and meta-analysis using the A Measurement Tool to Assess systematic Reviews (AMSTAR) 2 checklist (20–22). This is a standardized checklist including 16 criteria that refer to a corresponding methodological aspect of the study. We categorized the overall AMSTAR 2 grade as high, moderate, low, or extremely low quality.

### Evidence Grading

Using the methodology described earlier and according to the grading scheme applied in previously published studies, we classified the associations that presented nominally statistically significant summary results (*p* < 0.05) into convincing, highly suggestive, suggestive, weak evidence, or non-significant associations (23–26). Evidence was defined as convincing when the value of *p* of the random-effects model was smaller than 10^−6^, the meta-analysis included more than 1,000 cases or more than 20,000 participants for continuous outcomes if the largest component study in the meta-analysis reported a significant result (*p* < 0.05), if the 95% PIs excluded the null hypothesis if the *I*^2^ statistic for heterogeneity was <50% if there was no evidence of small-study effects (*p* > 0.10), and if excess significance bias (*p* > 0.10) was indicated.

Evidence was defined as highly suggestive if the value of *p* for the random-effects model was <10^−6^, if the meta-analysis included more than 1,000 cases or more than 20,000 participants for continuous outcomes, or if the largest component study reported a significant result. Evidence was defined as suggestive if the value of *p* for random effects was <10^−3^ or if there were more than 1,000 cases or more than 20,000 participants for continuous outcomes. Evidence was defined as weak if the value of *P* for significant associations was <0.05. We used the “non-significant associations” classification if all associations yielded the value of *p* > 0.05.

## Results

### Literature Review

**Figure 1:**
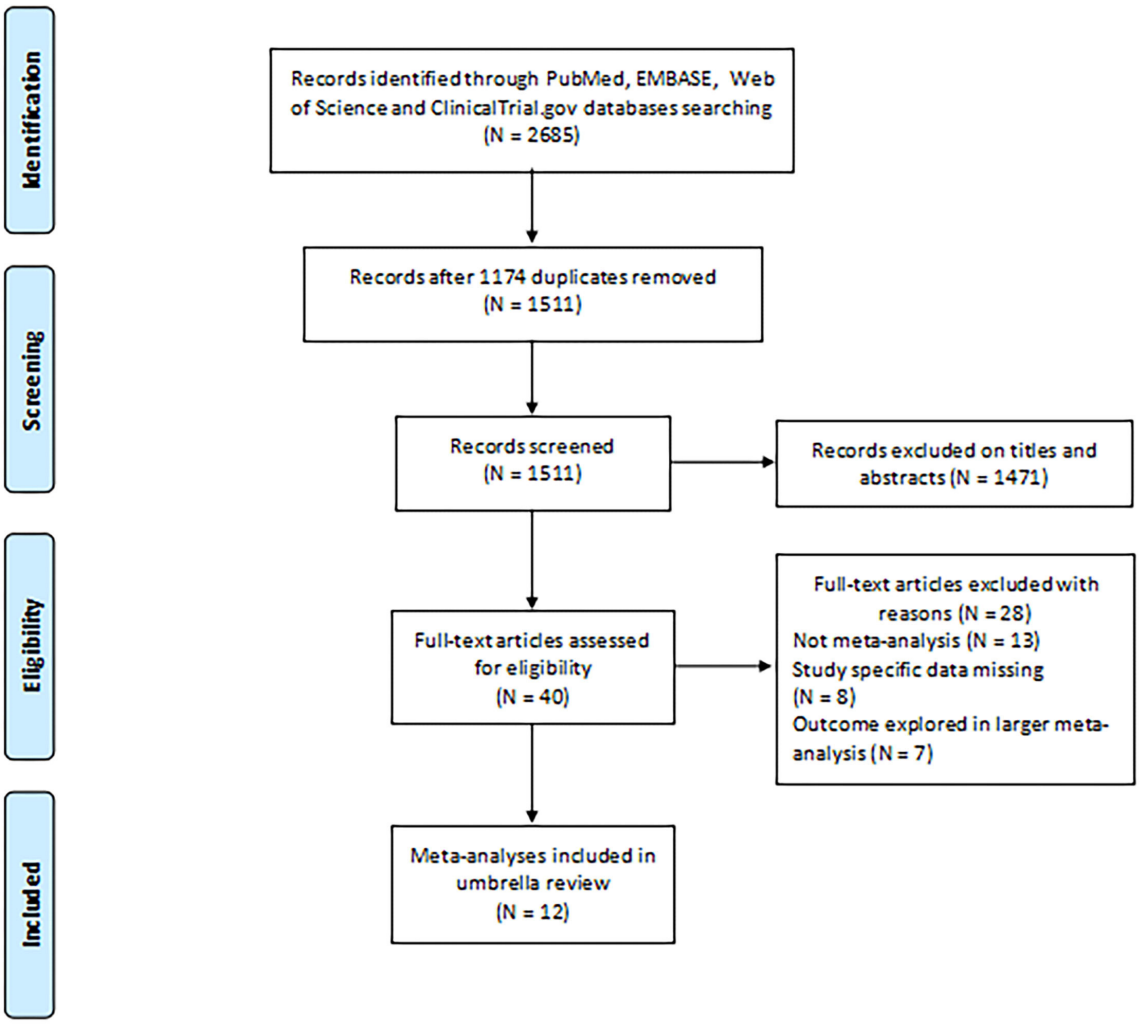
Flowchart depicting the selection of studies for inclusion in our umbrella review relating to the potential association between environmental risk factors and endometriosis.

As shown in Figure 1, our initial searches identified 2,685 potentially eligible articles from PubMed, EMBASE, and Web of Science. After applying the inclusion and exclusion criteria, 40 full texts were identified for analysis; of these, we selected 12 articles (9, 27–37) including 40 meta-analyses for the current umbrella review.

### Characteristics of the Meta-Analyses

As reported in Table 1 and Supplementary Table 4, the eligible meta-analyses were published between 2012 and 2020, and the median number of articles included for each risk factor was nine (range: 2–30). Of the 354 unique studies, 81 (22.9%) adopted cohort designs, 258 (72.9%) adopted a case-control design, and 15 (4.2%) were cross-sectional studies. About 40 associations between environmental risk factors and endometriosis were identified and based on data from 143,422 cases and a population of 5,112,967. A total of 26 associations included at least 1,000 cases of endometriosis. The meta-analyses reported a wide range of environmental risk factors related to lifestyles (*n* = 16), reproductive factors (*n* = 3), early life factors (*n* = 4), and a range of other risk factors, including vitamin D levels (*n* = 1), body mass index (BMI) (*n* = 3), an exposure to EDCs (*n* = 5), phthalate metabolites (*n* = 5), and race/ethnicity (*n* = 3).

**Table 1:** Characteristics and quantitative synthesis of the eligible meta-analyses of multiple risk factors for endometriosis.

**Risk factor (reference)**	**No. of studies**	**No. of cases/participants**	**Level of comparison**	**Summary relative risk (95% CI)**			**Random *p*-value^†^**	**Fixed *p*-value^‡^**
				**Random effects**	**Fixed effects**	Largest study^*^		
**Life style**								
Physical activity (9)	7	3,355/111,647	Any vs. no	0.85 (0.67–1.07)	0.98 (0.89–1.08)	1.05 (0.94–1.17)	0.17	0.72
	6	3,276/107,613	Low vs. no	1.00 (0.78–1.28)	1.03 (0.92–1.15)	1.04 (0.93–1.17)	0.98	0.60
	6	3,276/107,613	Moderate/high vs. no	0.75 (0.53–1.07)	0.74 (0.66–0.82)	0.89 (0.77–1.03)	0.11	4.35 × 10^−8^
Alcohol intake (24)	17	3,404/12,403	Any vs. no	1.25 (1.11–1.41)	1.24 (1.12–1.36)	1.01 (0.79–1.29)	2.70 × 10^−4^	2.13 × 10^−5^
	5	469/1,029	Infrequent vs. no	1.14 (0.86–1.52)	1.14 (0.86–1.52)	1.32 (0.82–2.13)	0.37	0.37
	11	1,813/7,994	Moderate/regular vs. no	1.27 (1.07–1.50)	1.23 (1.08–1.40)	1.00 (0.74–1.35)	0.005	0.002
	8	1,417/6,162	Heavy vs. no	1.19 (0.99–1.43)	1.19 (0.99–1.43)	1.01 (0.75–1.37)	0.07	0.07
Tobacco smoking (25)	24	9,616/821,028	Ever vs. never	0.96 (0.86–1.08)	1.05 (0.99–1.11)	1.20 (1.11–1.30)	0.53	0.09
	30	7,796/585,414	Current vs. never	0.92 (0.82–1.04)	0.96 (0.89–1.03)	1.20 (1.00–1.40)	0.18	0.24
	16	4,539/632,567	Former vs. never	0.95 (0.81–1.11)	0.92 (0.85–1.00)	0.90 (0.80–1.00)	0.51	0.06
	8	2,407/521,471	Moderate vs. never	0.87 (0.70–1.07)	0.87 (0.76–1.00)	1.00 (0.80–1.20)	0.20	0.05
	8	2,346/529,817	Heavy vs. never	0.93 (0.69–1.26)	1.18 (1.03–1.35)	1.35 (1.15–1.59)	0.64	0.02
Coffee intake (26)	3	387/772	Any vs. no	1.13 (0.46–2.76)	0.89 (0.70–1.14)	0.85 (0.66–1.10)	0.79	0.36
Caffeine intake (26)	5	1,020/196,119	Any vs. no	1.26 (0.95–1.66)	1.06 (0.96–1.17)	0.97 (0.87–1.09)	0.11	0.27
	5	1,178/197,650	High vs. no	1.09 (0.84–1.42)	1.03 (0.90–1.18)	0.95 (0.80–1.12)	0.51	0.65
	5	1,053/196,328	Low vs. no	1.09 (0.89–1.33)	1.05 (0.93–1.19)	1.00 (0.86–1.17)	0.41	0.43
**Reproductive factors**								
Early menarche (23)	18	3,805/13,331	Youngest vs. oldest	1.21 (0.99–1.47)	1.27 (1.15–1.40)	1.85 (1.44–2.39)	0.06	1.06 × 10^−6^
**Length of menstrual cycle**								
Menstrual cycle length SEQ27 (27)	5	954/4,251	Short vs. long	1.37 (1.04–1.80)	1.23 (1.05–1.44)	1.01 (0.82–1.24)	0.02	0.01
Menstrual cycle length LEQ29 (27)	4	454/1,354	Long vs. short	0.67 (0.48–0.96)	0.69 (0.55–0.88)	0.83 (0.58–1.18)	0.03	0.002
**Early life**								
Pre-term birth (33)	4	1,831/86,256	Exposed vs. unexposed	1.65 (1.07–2.53)	1.65 (1.07–2.53)	1.55 (0.93–2.51)	0.02	0.02
Low birth weight (33)	6	2,360/87,390	Exposed vs. unexposed	2.24 (1.36–3.67)	2.23 (1.43–3.50)	2.19 (1.07–4.47)	0.002	4.52 × 10^−4^
Diethylstilbestrol *in utero* (33)	2	1,536/85,483	Exposed vs. unexposed	4.49 (1.85–10.90)	4.49 (1.85–10.90)	4.47 (1.66–12.02)	0.001	0.001
Feeding pattern (33)	3	562/1,601	Breast feeding vs. formula feeding	3.63 (1.11–11.87)	4.54 (2.27–9.06)	8.91 (3.47–22.39)	0.03	1.82 × 10^−5^
**Other risk factors**								
Vitamin D levels (32)	8	547/1,151	Highest vs. lowest	0.17 (0.04–0.67)	0.36 (0.29–0.46)	0.88 (0.62–1.24)	0.01	5.31 × 10^−17^
Body mass index (28)	9	7,107/119,591	Each 5 kg/m^2^ increase in current BMI	0.67 (0.53–0.84)	0.93 (0.91–0.95)	0.95 (0.93–0.98)	0.001	3.16 × 10^−8^
	3	5,860/117,586	Obesity vs. normal	0.88 (0.67–1.17)	0.89 (0.83–0.96)	0.89 (0.82–0.96)	0.39	0.004
	5	8,107/120,971	Overweight vs. normal	0.97 (0.91–1.05)	0.97 (0.91–1.05)	0.99 (0.91–1.06)	0.48	0.48
**Endocrine-disrupting chemicals**								
EDCs (31)	30	2,551/8,622	Exposed vs. unexposed	1.41 (1.23–1.60)	1.15 (1.11–1.19)	1.01 (0.95–1.07)	3.93 × 10^−7^	3.00 × 10^−15^
PCBs (31)	12	1,055/3,396	Exposed vs. unexposed	1.58 (1.18–2.12)	1.16 (1.07–1.25)	0.96 (0.87–1.05)	0.002	1.41 × 10^−4^
OCPs (31)	8	668/1,900	Exposed vs. unexposed	1.40 (1.02–1.92)	1.23 (1.15–1.31)	0.91 (0.83–1.00)	0.04	1.39 × 10^−10^
PAEs (31)	6	431/2,196	Exposed vs. unexposed	1.27 (1.00–1.60)	1.07 (1.02–1.13)	1.01 (0.95–1.07)	0.048	0.009
BPA (31)	4	397/1,130	Exposed vs. unexposed	1.40 (0.94–2.08)	1.30 (1.13–1.50)	0.96 (0.79–1.19)	0.10	2.53 × 10^−4^
**Phthalate metabolites**								
MEHHP (30)	6	495/2,219	Exposed vs. unexposed	1.25 (1.00–1.55)	1.14 (1.01–1.28)	1.07 (0.88–1.21)	0.047	0.03
MEHP (30)	7	565/2,454	Exposed vs. unexposed	1.09 (0.86–1.38)	1.02 (1.00–1.04)	1.02 (1.00–1.04)	0.48	0.03
MEP (30)	6	468/2,188	Exposed vs. unexposed	1.07 (0.90–1.28)	1.07 (0.90–1.28)	1.01 (0.82–1.24)	0.43	0.43
MBzP (30)	7	523/2,276	Exposed vs. unexposed	0.98 (0.81–1.18)	0.98 (0.81–1.18)	0.84 (0.65–1.07)	0.80	0.80
MEOHP (30)	6	495/2,216	Exposed vs. unexposed	1.28 (0.87–1.88)	1.15 (0.96–1.39)	1.06 (0.85–1.32)	0.20	0.13
**Race/ethnicity**								
Race/ethnicity (29)	16	27,734/221,655	White vs. Black	0.49 (0.29–0.81)	0.19 (0.18–0.21)	0.12 (0.11–0.13)	0.006	0.00
Race/ethnicity (29)	10	6,273/107,840	White vs. Asian	1.63 (1.03–2.58)	1.16 (1.03–1.31)	0.76 (0.60–0.96)	0.04	0.01
Race/ethnicity (29)	5	21,292/80,283	White vs. Hispanic	0.46 (0.14–1.49)	0.16 (0.15–0.17)	0.15 (0.14–0.16)	0.20	0.00

*BPA, bisphenol A; EDCs, endocrine-disrupting chemicals; LEQ29, longer than or equal to 29 days; MEHHP, mono-(2-ethyl-5-hydroxyhexyl) phthalate; MEHP, mono (2-ethylhexyl) phthalate; MEP, monoethyl phthalate; MBzP, monobenzyl phthalate; MEOHP, mono (2-ethyl-5-oxohexyl) phthalate; OCPs, organochlorine pesticides; PCBs, polychlorinated biphenyls; PAEs, phthalate esters; SEQ27, shorter than or equal to 27 days*.

* *Relative risk (RR) and 95% CI of largest study (smallest SE) in each meta-analysis*.

† *p-value of summary random-effects estimate*.

‡ *p-value of summary fixed-effects estimate*.

*All statistical tests were two-sided*.

### Summary Effect Size

**Figure 2:**
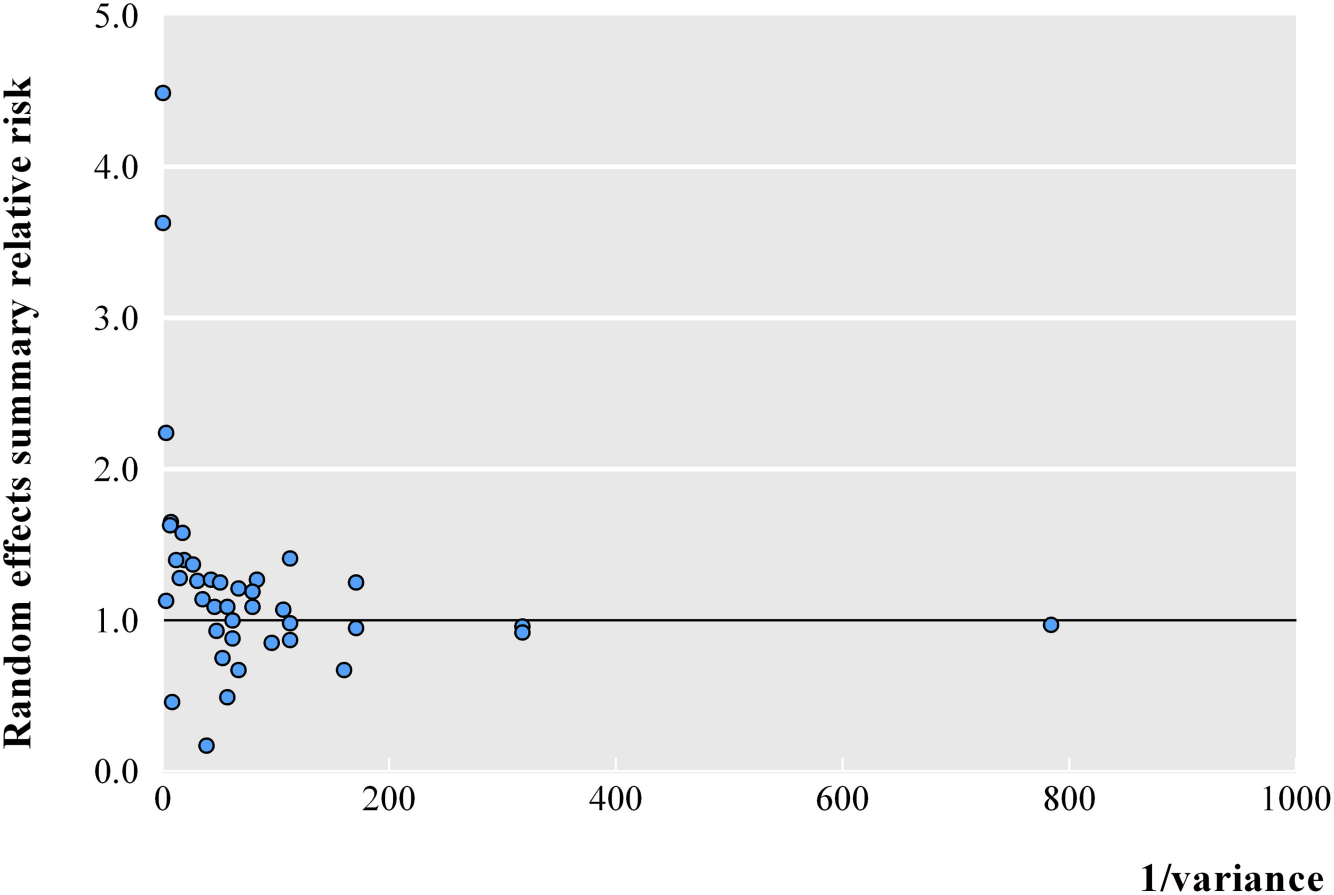
The association of meta-analysis summary effect sizes with the inverse of the variance.

At a threshold of *p* < 0.05, the summary effect sizes were significant for 25 (62.5%) and 17 (42.5%) association estimates in fixed- and random-effects models, respectively. At a more conservative threshold of *p* < 10^−6^, 7 (17.5%) and 1 (2.5%) associations were statistically significant in fixed- and random-effects models, respectively. The magnitude of the observed summary random effect estimates was in the range from 0.17 and 4.49, and 62.5% of the observed estimates were between 0.70 and 1.30 (Figure 2). In a meta-analysis with small variances, it was observed that the summary effect size tended to be 1. However, three associations exhibited evident outliers (feeding pattern in early life, diethylstilbestrol *in utero*, and BMI of overweight subjects vs. normal subjects).

### Heterogeneity and Bias Tests for Meta-Analyses

Overall, the largest study described statistically significant results in 15 meta-analyses (37.5%). About 12 meta-analyses (30.0%) exhibited large heterogeneity (*I*^2^ ≥ 50% and *I*^2^ ≤ 75%), and 11 (27.5%) showed very large heterogeneity (*I*^2^ > 75%) (Table 2). We further assessed the uncertainty of the summary random effects by calculating their 95% PIs; however, the 95% PI excluded the null hypothesis in none of the associations.

**Table 2:** Level of evidence for the association of risk factors for endometriosis.

**Risk factor (reference)**	**Features used for classification of level of evidence**								**Evidence class**
	Significance threshold reached^*^	***I*^**2**^ (95% CI)**	**95% prediction interval**	**Egger's *p*-value**	**Excess significance^§^**		**Largest study significant**	**Small-study effect/excess significant bias**	
					**O/E^#^**	***p*-value^¶^**			
**Life style**									
*Physical activity*									
Any physical activity (9)	>0.05	44.6% (0–77%)	0.47–1.52	0.12	1/1.52	NP	No	No/No	No association
Low level physical activity (9)	>0.05	30.3% (0–72%)	0.56–1.78	0.57	1/1.13	NP	No	No/No	No association
Moderate/high level physical activity (9)	>0.05	77.4% (50–90%)	0.28–2.01	0.92	1/1.67	NP	No	No/No	No association
*Alcohol intake*									
Any alcohol intake (24)	<0.001 but >10^−6^	23.5% (0–57%)	0.94–1.66	0.90	3/4.76	NP	No	No/No	Suggestive
Infrequent alcohol intake (24)	>0.05	0 (0–79%)	0.72–1.82	0.39	0/0.68	NP	No	No/No	No association
Moderate/regular alcohol intake (24)	<0.05 but >0.001	30.6% (0–66%)	0.86–1.87	0.03	2/3.19	NP	No	Yes/No	Weak
Heavy alcohol intake (24)	>0.05	0 (0–68%)	0.94–1.49	0.49	0/1.23	NP	No	No/No	No association
*Tobacco smoking*									
Ever smokers (25)	>0.05	63.8% (44–77%)	0.62–1.49	0.05	6/7.39	NP	Yes	Yes/No	No association
Current smokers (25)	>0.05	49.6% (23–67%)	0.59–1.44	0.09	3/9.45	NP	Yes	Yes/No	No association
Former smokers (25)	>0.05	51.0% (13–72%)	0.59–1.51	0.69	3/4.00	NP	Yes	No/No	No association
Moderate smokers (25)	>0.05	44.4% (0–75%)	0.51–1.49	0.96	1/1.41	NP	No	No/No	No association
Heavy smokers (25)	>0.05	59.3% (11–81%)	0.40–2.17	0.02	2/2.54	NP	Yes	Yes/No	No association
*Caffeine/coffee intake*									
Any coffee intake (26)	>0.05	70.0% (0–91%)	0–28979.81	0.69	1/0.85	1	No	No/No	No association
Any caffeine intake (26)	>0.05	67.6% (16–87%)	0.52–3.05	0.20	1/1.69	NP	No	No/No	No association
High caffeine intake (26)	>0.05	57.3% (0–84%)	0.49–2.44	0.72	1/1.41	NP	No	No/No	No association
Low caffeine intake (26)	>0.05	36.8% (0–76%)	0.63–1.88	0.45	1/0.997	1	No	No/No	No association
**Reproductive factors**									
Early menarche (23)	>0.05	72.2% (55–83%)	0.57–2.56	0.25	6/9.13	NP	Yes	No/No	No association
*Length of menstrual cycle*									
Menstrual cycle length SEQ27 (27)	<0.05 but >0.001	53.1% (0–83%)	0.60–3.15	0.12	2/1.82	1	No	No/No	Weak
Menstrual cycle length LEQ29 (27)	<0.05 but >0.001	48.4% (0–83%)	0.18–2.47	0.68	1/1.12	NP	No	No/No	Weak
**Early life**									
Pre-term birth (33)	<0.05 but >0.001	0 (0–85%)	0.64–4.22	0.87	0/0.37	NP	No	No/No	Weak
Low birth weight (33)	<0.05 but >0.001	12.8% (0–78%)	0.87–5.74	0.94	2/0.98	0.26	Yes	No/No	Weak
Diethylstilbestrol *in utero* (33)	<0.05 but >0.001	0	NA	NA	1/0.10	0.098	Yes	No / Yes	Weak
Feeding pattern (33)	<0.05 but >0.001	61.9% (0–89%)	0–1569934.50	0.20	1/1.09	NP	Yes	No/No	Weak
**Other risk factors**									
Vitamin D levels (32)	<0.05 but >0.001	96.2% (94–97%)	0.00–23.98	0.258	4/3.87	1	No	No/No	Weak
*Body mass index*									
Body mass index (each 5 kg/m^2^ increase) (28)	<0.05 but >0.001	87.0% (77–93%)	0.31–1.47	<0.01	6/5.10	0.74	Yes	Yes/No	Weak
Body mass index (Obesity) (28)	>0.05	50.1% (0–86%)	0.05–15.57	0.72	2/0.62	0.11	Yes	No / Yes	No association
Body mass index (Overweight) (28)	>0.05	0.0 (0–79%)	0.87–1.10	0.04	0/0.71	NP	No	Yes/No	No association
*Endocrine–disrupting chemicals*									
EDCs (31)	<10^−6^	88.7% (85–91%)	0.76–2.60	<0.01	16/14.74	0.72	No	Yes/No	Suggestive
PCBs (31)	<0.05 but >0.001	84.3% (74–90%)	0.62–4.07	0.05	5/5.43	NP	No	Yes/No	Weak
OCPs (31)	<0.05 but >0.001	94.3% (91–96%)	0.48–4.09	0.42	6/5.04	0.72	Yes	No/No	Weak
PAEs (31)	<0.05 but >0.001	84.5% (70–93%)	0.58–2.77	0.17	4/2.85	0.43	No	No/No	Weak
BPA (31)	>0.05	81.6% (52–93%)	0.25–7.74	0.73	1/1.76	NP	No	No/No	No association
*Phthalate metabolites*									
MEHHP (30)	<0.05 but >0.001	44.1% (0–78%)	0.72–2.15	0.08	2/1.40	0.63	No	Yes/No	Weak
MEHP (30)	>0.05	59.2% (6–82%)	0.60–1.98	0.64	2/2.05	NP	Yes	No/No	No association
MEP (30)	>0.05	0 (0–75%)	0.83–1.38	0.35	0/0.94	NP	No	No/No	No association
MBzP (30)	>0.05	0 (0–71%)	0.76–1.25	0.02	0/1.28	NP	No	Yes/No	No association
MEOHP (30)	>0.05	54.1% (0–82%)	0.44–3.76	0.48	2/1.58	0.66	No	No/No	No association
*Race/ethnicity*									
White vs. Black (29)	<0.05 but >0.001	96.5% (95–97%)	0.06–4.01	<0.01	7/13.00	NP	Yes	Yes/No	Weak
White vs. Asian (29)	<0.05 but >0.001	90.2% (84–94%)	0.35–7.61	0.16	6/4.86	0.54	Yes	No/No	Weak
White vs. Hispanic (29)	>0.05	94.1% (89–97%)	0.01–30.72	0.17	1/2.24	NP	Yes	No/No	No association

*BPA, bisphenol A; EDCs, endocrine-disrupting chemicals; LEQ29, longer than or equal to 29 days; MEHHP, mono-(2-ethyl-5-hydroxyhexyl) phthalate; MEHP, mono (2-ethylhexyl) phthalate; MEP, monoethyl phthalate; MBzP, monobenzyl phthalate; MEOHP, mono (2-ethyl-5-oxohexyl) phthalate; NA, not available, due to <3 included studies; NP, not pertinent, because the estimated is larger than the observed, and there is no evidence of excess statistical significance based on the assumption made for the plausible effect size; OCPs, organochlorine pesticides; PCBs, polychlorinated biphenyls; PAEs, phthalate esters; SEQ27, shorter than or equal to 27 days*.

§ *Expected number of statistically significant studies using the point estimate of the largest study (smallest SE) as the plausible effect size*.

# *Observed/expected number of statistically significant studies*.

¶ *p-value of the excess statistical significance test*.

* *p-value under the random-effects model*.

Evidence for significant small-study effects was observed in 11 meta-analyses, and evidence for statistically significant excess significance bias was noted for the one risk factors (diethylstilbestrol *in utero*).

### The Methodological Quality of the Meta-Analyses

After evaluating the risk of bias using the AMSTAR 2 tool, the conduct of the articles was rated as low quality for 33.3% (*n* = 4) of the published articles, and critically low for 66.7% (*n* = 8) (Supplementary Figure 1). The most frequently deficient critical domains were the lack of a registered protocol (9 of 12 articles) and the lack of a list of excluded studies or justification of the exclusions (9 of 12 articles).

### Grading of the Evidence

By applying the predefined methodological criteria, we further investigated whether the nominally significant associations between environmental risk factors and endometriosis were supported by convincing evidence, highly suggestive evidence, suggestive evidence, weak evidence, or no association (Table 2). Overall, no association was supported through convincing and highly suggestive evidence, whereas the associations of the two risk factors (any alcohol intake and exposure to EDCs) for endometriosis were supported by suggestive evidence by virtue of the fact that these two risk factors involved >1,000 cases and the value of *p* for random effects <10^−3^. Furthermore, 15 associations (moderate/regular alcohol intake, the length of menstrual cycle [shorter than or equal to 27 days (SEQ27) and longer than or equal to 29 days (LEQ29)], pre-term birth, low birth weight, diethylstilbestrol *in utero*, feeding pattern, vitamin D levels, BMI, polychlorinated biphenyls (PCBs), organochlorine pesticides (OCPs), phthalate esters (PAEs), mono-(2-ethyl-5-hydroxyhexyl) phthalate (MEHHP), and race/ethnicity [white vs. black or Asian]) presented weak evidence with the value of *p* for random effects <0.05. Finally, 23 associations did not present even a nominally statistically significant result.

## Discussion

This umbrella review involves previous meta-analyses of observational studies and provides a comprehensive overview and critical assessment of the environmental risk factors associated with endometriosis. About 40 risk factors were investigated for their association with endometriosis, including lifestyle, reproductive factors, early life factors, race/ethnicity, and other risk factors. However, among these factors, only the two factors (any alcohol intake and EDCs) presented suggestive evidence to indicate a strong, significant, and positive association with endometriosis. Several other putative risk factors (e.g., moderate/regular alcohol intake, the length of menstrual cycle, and early life factors such as, pre-term birth, low birth weight, and feeding pattern) presented weak evidence for their association with endometriosis.

Four of the included meta-analyses investigated the relationship between alcohol intake and the risk of endometriosis (9). However, only the “any alcohol intake” criterion was supported by evidence with suggestive epidemiological credibility. Furthermore, despite a significant positive relationship between any alcohol intake and the risk of endometriosis, the 95% PI of the effect size included the null hypothesis, thus showing that in some settings the effect of any alcohol intake on endometriosis might be absent. Consistent with our findings, a review conducted by Agarwal et al. (38) reported that alcohol consumption may catalyze the production of oxidative stress and reactive oxygen species; these factors may increase the risk of endometriosis (38). Endometriosis is an estrogen-dependent disease (39), and ovarian sex steroid receptors have been identified in ectopic endometrial tissue (40). Women suffering from alcohol dependence or abuse are often anovulatory and exhibit ovarian pathology, luteal phase defects, recurrent abortion, and infertility (41). Occasionally, alcoholics have premenstrual symptoms, dysmenorrhea, and a heavy menstrual flow (41). All of these factors are known to be related to endometriosis (41). It is plausible that alcohol could increase the activity of aromatase, an enzyme that converts testosterone to estrogen, thus leading to a reduction in testosterone levels and an increase in estrogen levels (42). Alcohols may also interact with a luteinizing hormone derived from the pituitary, thus causing the ovaries to release more amount of estradiol (43). The summary RR was significant and showed a relatively strong effect between the exposure to EDCs and the risk of endometriosis with suggestive epidemiological evidence. However, this particular meta-analysis (35) exhibited very large levels of heterogeneity, small-study effects; moreover, the 95% PI included the null hypothesis, thus showing that the effect size of the relationship might vary in different settings.

Endocrine disruptor chemicals are exogenous chemical entities or mixtures of compounds, which exert their toxicity by interfering with the normal hormonal homeostatic mechanisms that promote the growth and development of tissues (44). Our results are consistent with several previous experimental studies, which reported that the exposure of prenatal mice to bisphenol A (BPA) can cause endometriosis-like symptoms in offspring (45, 46). The relationship between EDCs and the risk of endometriosis is credible because EDCs exhibit a variety of biological effects, including the ability to alter hormone synthesis, regulate receptors, or act as agonists or antagonists (47). Estrogen is necessary for the proliferation and survival of endometriotic tissues (48). A study conducted by Lemaire et al. (49) indicated that many EDCs bind and activate estrogen receptor- (ER-) alpha and exhibit a dose-dependent agonist/antagonist effect on ER signaling; this effect is essential for angiogenesis and inflammatory signaling during the development of endometriotic lesions.

Additional potential risk factors showed weak evidence for endometriosis, including moderate/regular alcohol intake, the length of the menstrual cycle, early life factors (e.g., pre-term birth, low birth weight, and feeding pattern) vitamin D levels, BMI, certain types of EDCs (e.g., PCBs and OCPs), MEHHP, and race/ethnicity factors. This might be due to the fact that these meta-analyses had large or very large levels of heterogeneity and small-study effect/excess significant bias. We also found that breastfeeding was positively associated with the risk of endometriosis although this may be affected by a certain amount of bias (37). A previous experimental study involving Wistar rats described the evolution of endometriotic implants from pregnancy to lactation and showed a marked tendency for regression in the grade of growth (50). Histologically, during lactation, the endometriotic implants showed signs of reduced cellular activity, such as the presence of tiny cysts in the epithelium-lining that were devoid of vesicular nuclei or prominent nucleoli with only a small extent of apical cytoplasmic secretion (50). To some extent, this suggests that breastfeeding may reduce the risk of endometriosis. This might be due to the fact that breastfeeding can prolong post-partum amenorrhea, influence retrograde menstruation, increase concentrations of circulating oxytocin, and inhibit circulating concentrations of estrogen, gonadotrophin-releasing hormone, luteinizing hormone, and follicle-stimulating hormone (51).

With regard to race/ethnicity, the results related to the risk of endometriosis have been inconsistent thus far. For example, a meta-analysis conducted by Bougie indicated that the risk of endometriosis is higher in Asian women and lower in black women (33); this was contradictory to our present findings. This might be related to socioeconomic factors. When the unit of analyses was a group, race/ethnicity was kept constant, the socioeconomic level of the entire study population was regarded as homogeneous, the prevalence of endometriosis was positively associated with socioeconomic status (52). In another paper, Ridley stated that the frequency of endometriosis increases in any racial group as socioeconomic status improves. It would be expected that women with a higher socioeconomic status in more affluent countries may have better access to care and therefore would be more likely to be diagnosed.

To the best of our knowledge, this is the first systematic and comprehensive assessment of the potential association between environmental factors and the risk of endometriosis using a robust analysis and by evaluating biases and methodological limitations. The classification of evidence was based on extensive statistical analysis and a large number of previous meta-analyses and aims to assess the strength and validity of the published evidence. The criteria chosen to classify each meta-analysis by evidence level (i.e., convincing, highly suggestive, suggestive, or weak) is a transparent and systematic means of assessing the strength of evidence in the literature. In addition, we used AMSTAR-2 to assess the methodological quality of the mate-analyses included; this strategy had helped to identify the most common reasons for reduced quality and will help to improve the quality of future articles in this field.

Nevertheless, there are some possible limitations and caveats associated with this study. First, the present analysis relies upon the articles cited by the original authors and the results that have already been published in systematic reviews and meta-analyses. Although some studies may have been missed in the initial searches, this is unlikely to have affected our results, as repeat meta-analysis resulted in similar results. Second, the statistical tests we used to explore the existence of bias can only provide clues for the presence of potential bias, these tests cannot prove the existence of bias or its exact source. However, our estimates may be conservative because a negative result for bias does not rule out its potential existence. Third, we did not assess the quality of the preliminary studies for individual components as this was beyond the scope of this umbrella review; rather, the initial systematic review and meta-analysis were responsible for this aspect. However, we classified epidemiological evidence on the basis of well-recognized and pre-specified criteria. Fourth, some of the included meta-analyses exhibited large or very large levels of heterogeneity, along with signs of small-study effects or excess significance. In cases of particularly extensive heterogeneity, it is possible that Egger's test may lead to false signals with small-study effects (53, 54). Heterogeneity may often be an expression of bias in some studies involving meta-analysis although this can also arise from real discrepancies between different studies. To our knowledge, the incidence and prevalence of endometriosis show obvious geographical heterogeneity; this may manifest as risk factors showing differential relationships in different geographical regions (2). In addition, there are several other factors that could contribute to heterogeneity, including the integration of cohort studies and case-control studies, discrepancies in the estimation of exposure, discrepancies in the definition and diagnosis of endometriosis, the frequency of exposure in control groups, the types of exposure and the source of controls, and differential response rates among cases and controls. Therefore, we should consider the association between environmental factors and risk factors for endometriosis with caution, particularly for meta-analyses exhibiting extensive heterogeneity. Finally, while we are concerned about bias and other issues that may lead to false-positive relationships, it is also that false negatives may also exist, particularly for associations with limited evidence. Despite these potential limitations, we describe the state of association between environmental factors and the risk of endometriosis. The potential clinical significance of identifying strong correlations between these parameters is to identify individuals at a higher risk of endometriosis. This may allow us to organize appropriate screening programs to detect the preclinical phases of endometriosis.

## Conclusion

In conclusion, the intake of any alcohol and exposure to EDCs may represent potential risk factors for endometriosis; these associations were linked through suggestive epidemiological evidence. However, further research is still needed. Such research should incorporate data from a larger number of studies and investigate the specific sources of heterogeneity so that we can better understand the relationship between these factors and the risk of endometriosis.

## Data Availability Statement

The data that support the findings of this study are available from the corresponding author upon reasonable request. Requests to access these datasets should be directed to maningyehead@163.com.

## Author Contributions

N-YM: conceived and designed the study. YZ: literature search, data curation, formal analysis, and writing the original draft. All authors: writing, reviewing, and editing.

## Conflict of Interest

The authors declare that the research was conducted in the absence of any commercial or financial relationships that could be construed as a potential conflict of interest.

## Publisher's Note

All claims expressed in this article are solely those of the authors and do not necessarily represent those of their affiliated organizations, or those of the publisher, the editors and the reviewers. Any product that may be evaluated in this article, or claim that may be made by its manufacturer, is not guaranteed or endorsed by the publisher.
