# Supplementary material for: Environmental Risk Factors for Endometriosis: An Umbrella Review of a Meta-Analysis of 354 Observational Studies With Over 5 Million Populations
Source: Front Med (Lausanne). 2021 Oct 25;8:680833. doi: 10.3389/fmed.2021.680833 (PMC8573094; doi:10.3389/fmed.2021.680833)
Supplement: Supplementary file 1 [file Data_Sheet_1.docx]

**Supplementary table S1. PRISMA 2009 Checklist.**

| **Section/topic** | **#** | **Checklist item** | **Reported on page #** |
| --- | --- | --- | --- |
| **TITLE** | | |  |
| Title | 1 | Identify the report as a systematic review, meta-analysis, or both. | 1 |
| **ABSTRACT** | | |  |
| Structured summary | 2 | Provide a structured summary including, as applicable: background; objectives; data sources; study eligibility criteria, participants, and interventions; study appraisal and synthesis methods; results; limitations; conclusions and implications of key findings; systematic review registration number. | 2-3 |
| **INTRODUCTION** | | |  |
| Rationale | 3 | Describe the rationale for the review in the context of what is already known. | 4-5 |
| Objectives | 4 | Provide an explicit statement of questions being addressed with reference to participants, interventions, comparisons, outcomes, and study design (PICOS). | 4-5 |
| **METHODS** | | |  |
| Protocol and registration | 5 | Indicate if a review protocol exists, if and where it can be accessed (e.g., Web address), and, if available, provide registration information including registration number. | PROSPERO (number: CRD42020200094) |
| Eligibility criteria | 6 | Specify study characteristics (e.g., PICOS, length of follow-up) and report characteristics (e.g., years considered, language, publication status) used as criteria for eligibility, giving rationale. | 6-7 |
| Information sources | 7 | Describe all information sources (e.g., databases with dates of coverage, contact with study authors to identify additional studies) in the search and date last searched. | 5-6 |
| Search | 8 | Present full electronic search strategy for at least one database, including any limits used, such that it could be repeated. | 5-6  Supplementary Table S3 |
| Study selection | 9 | State the process for selecting studies (i.e., screening, eligibility, included in systematic review, and, if applicable, included in the meta-analysis). | 6-7 |
| Data collection process | 10 | Describe method of data extraction from reports (e.g., piloted forms, independently, in duplicate) and any processes for obtaining and confirming data from investigators. | 7 |
| Data items | 11 | List and define all variables for which data were sought (e.g., PICOS, funding sources) and any assumptions and simplifications made. | 6-7 |
| Risk of bias in individual studies | 12 | Describe methods used for assessing risk of bias of individual studies (including specification of whether this was done at the study or outcome level), and how this information is to be used in any data synthesis. | 8-9 |
| Summary measures | 13 | State the principal summary measures (e.g., risk ratio, difference in means). | 8 |
| Synthesis of results | 14 | Describe the methods of handling data and combining results of studies, if done, including measures of consistency (e.g., I^2^) for each meta-analysis. | 8 |

| **Section/topic** | **#** | **Checklist item** | **Reported on page #** |
| --- | --- | --- | --- |
| Risk of bias across studies | 15 | Specify any assessment of risk of bias that may affect the cumulative evidence (e.g., publication bias, selective reporting within studies). | 8-9 |
| Additional analyses | 16 | Describe methods of additional analyses (e.g., sensitivity or subgroup analyses, meta-regression), if done, indicating which were pre-specified. | 8-10 |
| **RESULTS** | | |  |
| Study selection | 17 | Give numbers of studies screened, assessed for eligibility, and included in the review, with reasons for exclusions at each stage, ideally with a flow diagram. | 10-11  Figure 1 |
| Study characteristics | 18 | For each study, present characteristics for which data were extracted (e.g., study size, PICOS, follow-up period) and provide the citations. | 11  Table 1  Supplementary Table S4 |
| Risk of bias within studies | 19 | Present data on risk of bias of each study and, if available, any outcome level assessment (see item 12). | 12-13  Table2 |
| Results of individual studies | 20 | For all outcomes considered (benefits or harms), present, for each study: (a) simple summary data for each intervention group (b) effect estimates and confidence intervals, ideally with a forest plot. | 12  Table 1 |
| Synthesis of results | 21 | Present results of each meta-analysis done, including confidence intervals and measures of consistency. | 11-12  Table 1 |
| Risk of bias across studies | 22 | Present results of any assessment of risk of bias across studies (see Item 15). | 12  Table2 |
| Additional analysis | 23 | Give results of additional analyses, if done (e.g., sensitivity or subgroup analyses, meta-regression [see Item 16]). | 12-13 |
| **DISCUSSION** | | |  |
| Summary of evidence | 24 | Summarize the main findings including the strength of evidence for each main outcome; consider their relevance to key groups (e.g., healthcare providers, users, and policy makers). | 13-17 |
| Limitations | 25 | Discuss limitations at study and outcome level (e.g., risk of bias), and at review-level (e.g., incomplete retrieval of identified research, reporting bias). | 17-19 |
| Conclusions | 26 | Provide a general interpretation of the results in the context of other evidence, and implications for future research. | 19 |
| **FUNDING** | | |  |
| Funding | 27 | Describe sources of funding for the systematic review and other support (e.g., supply of data); role of funders for the systematic review. | None |
|  |  |  |  |

*From:*  Moher D, Liberati A, Tetzlaff J, Altman DG, The PRISMA Group (2009). Preferred Reporting Items for Systematic Reviews and Meta-Analyses: The PRISMA Statement. PLoS Med 6(6): e1000097. doi:10.1371/journal.pmed1000097

**Supplementary table S2. MOOSE Checklist for Meta-analyses of Observational Studies**

| **Item No** | **Recommendation** | **Reported on Page No** |
| --- | --- | --- |
| Reporting of background should include | | |
| 1 | Problem definition | 4 |
| 2 | Hypothesis statement | 4 |
| 3 | Description of study outcome(s) | 4 |
| 4 | Type of exposure or intervention used | 5 |
| 5 | Type of study designs used | 5 |
| 6 | Study population | 5 |
| Reporting of search strategy should include | | |
| 7 | Qualifications of searchers (eg, librarians and investigators) | 5-6 |
| 8 | Search strategy, including time period included in the synthesis and key words | 5-6  Supplementary Table S3 |
| 9 | Effort to include all available studies, including contact with authors | 5-6 |
| 10 | Databases and registries searched | 5-6 |
| 11 | Search software used, name and version, including special features used (eg, explosion) | 6 |
| 12 | Use of hand searching (eg, reference lists of obtained articles) | 6 |
| 13 | List of citations located and those excluded, including justification | 6-7 |
| 14 | Method of addressing articles published in languages other than English | 6-7  Figure1 |
| 15 | Method of handling abstracts and unpublished studies | 6-7  Figure1 |
| 16 | Description of any contact with authors | N/A |
| Reporting of methods should include | | |
| 17 | Description of relevance or appropriateness of studies assembled for assessing the hypothesis to be tested | 6-7 |
| 18 | Rationale for the selection and coding of data (eg, sound clinical principles or convenience) | 7 |
| 19 | Documentation of how data were classified and coded (eg, multiple raters, blinding and interrater reliability) | 7 |
| 20 | Assessment of confounding (eg, comparability of cases and controls in studies where appropriate) | 8-10 |
| 21 | Assessment of study quality, including blinding of quality assessors, stratification or regression on possible predictors of study results | 9 |
| 22 | Assessment of heterogeneity | 8 |
| 23 | Description of statistical methods (eg, complete description of fixed or random effects models, justification of whether the chosen models account for predictors of study results, dose-response models, or cumulative meta-analysis) in sufficient detail to be replicated | 8-10 |
| 24 | Provision of appropriate tables and graphics | Figure1  Supplementary figure S1 |
| Reporting of results should include | | |
| 25 | Graphic summarizing individual study estimates and overall estimate | 10-13 |
| 26 | Table giving descriptive information for each study included | Table 1,  Supplementary Table S4 |
| 27 | Results of sensitivity testing (eg, subgroup analysis) | None |
| 28 | Indication of statistical uncertainty of findings | None |

| **Item No** | **Recommendation** | **Reported on Page No** |
| --- | --- | --- |
| Reporting of discussion should include | | |
| 29 | Quantitative assessment of bias (eg, publication bias) | 17-19 |
| 30 | Justification for exclusion (eg, exclusion of non-English language citations) | None |
| 31 | Assessment of quality of included studies | 14-17  Supplementary figure S4 |
| Reporting of conclusions should include | | |
| 32 | Consideration of alternative explanations for observed results | 19 |
| 33 | Generalization of the conclusions (ie, appropriate for the data presented and within the domain of the literature review) | 19 |
| 34 | Guidelines for future research | 19 |
| 35 | Disclosure of funding source | None |

*From*: Stroup DF, Berlin JA, Morton SC, et al, for the Meta-analysis Of Observational Studies in Epidemiology (MOOSE) Group. Meta-analysis of Observational Studies in Epidemiology. A Proposal for Reporting. *JAMA*. 2000;283(15):2008-2012. doi: 10.1001/jama.283.15.2008.

Transcribed from the original paper within the NEUROSURGERY® Editorial Office, Atlanta, GA, United Sates. August 2012.

**Supplementary table S3. Search strategy used in the umbrella review**

| **Literature search strategy in PubMed** |
| --- |
| 1: endometriosis  2: meta-analysis OR systematic review  3: 1 AND 2 |
| **Literature search strategy in EMBASE** |
| 1: endometriosis  2: ‘meta analysis’ OR ‘systematic review’  3: 1 AND 2 |
| **Literature search strategy in Web of Science** |
| 1: Search TS= (endometriosis)  2: Search TS= (meta-analysis OR systematic review)  3: 1 AND 2 |

**Supplementary table S4.** **Description of the 12 meta-analysis of** **risk factors for endometriosis.**

| **Author** | **Year** | **Journal** | **Population** | **Study design included in meta-analysis** | **Risk factor** | **Type of**  **effect metric** |
| --- | --- | --- | --- | --- | --- | --- |
| Nnoaham^23^ | 2012 | Fertility and Sterility | Mixed | Case-control | Early menarche | SMD |
| Parazzini^24^ | 2013 | American Journal of Obstetrics & Gynecology | Mixed | Cohort and Case-control | Alcohol intake | RR |
| Chiaffarino^26^ | 2014 | European Journal of Nutrition | General | Cohort and Case-control | Coffee and caffeine intake | RR |
| Bravi^25^ | 2014 | BMJ Open | Mixed | Cohort and Case-control | Tobacco smoking | RR |
| Wei^27^ | 2016 | Medicine | Mixed | Case-control | Length of menstrual cycle | OR |
| Ricci^28^ | 2016 | Medicine | Mixed | Cohort and Case-control | Physical activity | OR |
| Liu^29^ | 2017 | Oncotarget | General | Cohort and Case-control | Body mass index | RR |
| Wen^32^ | 2019 | Gynecological Endocrinology | Mixed | Cohort, Case-control, and Cross-sectional | EDCs, PCBs, OCPs, PAEs, and BPA | RR |
| Cai^31^ | 2019 | International Journal of Environmental Research and Public Health | Mixed | Cohort, Case-control, and Cross-sectional | Five phthalate metabolites (MEHHP, MEHP, MEP, MBzP, and MEOHP) | OR |
| Bougie^30^ | 2019 | BJOG | Mixed | Cohort, Case-control, and Cross-sectional | Race/ethnicity | OR |
| Qiu^33^ | 2020 | Archives of Gynecology and Obstetrics | Mixed | Case-control | Vitamin D levels | OR |
| Ottolina^34^ | 2020 | Reproductive BioMedicine Online | Not report | Cohort and Case-control | Preterm birth, low birth weight, diethylstilbestrol in utero, and feeding | OR |

Abbreviations: BPA: bisphenol A; EDCs: Endocrine-disrupting chemicals; MEHHP: mono-(2-ethyl-5-hydroxyhexyl) phthalate; MEHP: mono (2-ethylhexyl) phthalate; MEP: monoethyl phthalate; MBzP: monobenzyl phthalate; MEOHP: mono (2-ethyl-5-oxohexyl) phthalate; OCPs: organochlorine pesticides; OR: odds ratio; PCBs: polychlorinated biphenyls; PAEs: phthalate esters; RR: relative risk；SMD：standardized mean difference

**Supplementary figure S1. AMSTAR2 rating of the included systematic reviews and meta-analyses.**


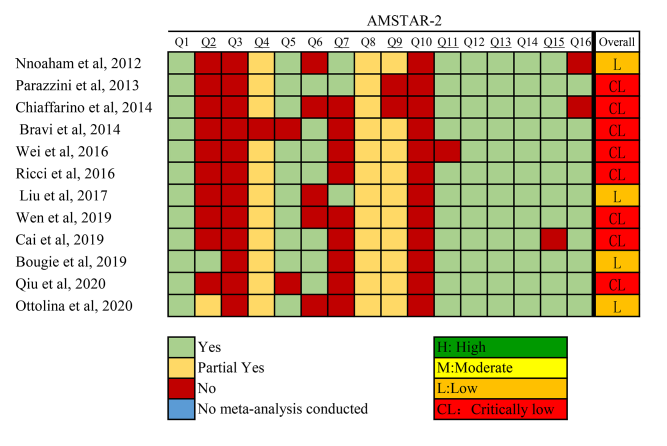


Q = Question; AMSTAR 2 critical domains included Q2, Q4, Q7, Q9, Q11, Q13, and Q15. Details about the AMSTAR 2 can be found elsewhere (doi: 10.1136/bmj.j4008).
